# Supplementary material for: Self-care interventions to reduce, prevent or monitor physical disability in those affected by leprosy: Protocol for a systematic review
Source: PLoS One. 2025 Oct 30;20(10):e0330477. doi: 10.1371/journal.pone.0330477 (PMC12574909; doi:10.1371/journal.pone.0330477)
Supplement: S2 File — (DOCX) [file pone.0330477.s002.docx]

# **SI 1** Table 1 Search Strategy

Sample search date 27/01/2025

PubMed

| Number | Search | Results |
| --- | --- | --- |
| #1 | "leprosy"[MeSH Terms] OR "hansen’s disease"[Title/Abstract] OR "hansens disease"[Title/Abstract] OR "Leprosy"[Title/Abstract] | 28,054 |
| #2 | "Self Care"[MeSH Terms] OR “Selfcare”[Title/abstract] OR "Self care"[Title/Abstract] OR "Self-care"[Title/Abstract] OR "self management"[Title/Abstract] OR “self-management"[Title/Abstract] OR "self-management"[Title/Abstract] OR "self management"[Title/Abstract] OR "self-treatment"[Title/Abstract] OR "self treatment "[Title/Abstract] OR "self-efficacy"[Title/Abstract] OR "self efficacy"[Title/Abstract] OR "peer support"[Title/Abstract] OR “patient empower*”[Title/Abstract] OR “Self Examination”[Title/Abstract] OR “Self-examination” [Title/Abstract] OR “self-guided care” [Title/Abstract] | 158,582 |
| #4 | #1 AND #2 | 142 |

Medline ALL

| Number | Search | Results |
| --- | --- | --- |
| #1 | Hansen's Disease.mp. or Leprosy/ or Leprosy.mp. OR Hansens Disease.mp. | 28,056 |
| #2 | Self Care.mp. OR Self care/ OR Self-care.mp. OR selfcare.mp.OR self management OR self-management.mp. OR "self-management.mp OR self management.mp. OR self-treatment.mp. OR self treatment.mp. OR self-efficacy.mp. OR self efficacy.mp. OR peer support.mp. OR “patient empower*.mp. OR Self Examination.mp. OR Self-examination.mp. OR self-guided care.mp. | 81,874 |
| #4 | #1 AND #2 | 96 |

Web of science Core Collection

| Number | Search | Results |
| --- | --- | --- |
| #1 | (TS=(leprosy)) OR TS=(Hansen’s disease) OR TS=(Hansens disease) | 17,873 |
| #2 | (TS=(Self Care) OR TS=(Self care) OR TS=(Self-care) OR TS=(self management) OR TS=(self-management) OR TS=(self-management) OR TS=(self management) OR TS=(self-treatment) OR TS=(self treatment) OR TS=(self-efficacy) OR TS=(self efficacy) OR TS=(peer support) OR TS=(patient empower*) OR TS=(Self Examination) OR TS=(Self-examination) OR TS=(self-guided care) | 730,417 |
| #4 | #1 AND #2 | 258 |

EMBASE

| Number | Search | Results |
| --- | --- | --- |
| #1 | Exp leprosy/ OR hansen’s disease.ti,ab,kf OR hansens disease.ti,ab,kf Or Leprosy.ti,ab,kf | 29,989 |
| #2 | Exp Self Care/ OR Self-care.ti,ab,kf OR Self care.ti,ab,kf OR Selfcare.ti,ab,kf OR self management.ti,ab,kf OR self-management.ti,ab,kf OR self-treatment.ti,ab,kf OR self treatment.ti,ab,kf OR self-efficacy.ti,ab,kf OR self efficacy.ti,ab,kf OR peer support.ti,ab,kf OR patient empower*.ti,ab,kf OR Self Examination.ti,ab,kf OR Self-examination.ti,ab,kf OR self-guided care.ti,ab,kf | 196,678 |
| #4 | #1 AND #2 | 223 |

LILACS spanish

| Number | Search | Results |
| --- | --- | --- |
| #1 | lepra OR enfermedad de Hansen OR hanseniasis | 447.373 |
| #2 | Autocuidado OR cuidado personal OR autoayuda | 46.867 |
| #4 | Lepra OR enfermedad de Hansen OR hanseniasis AND Autocuidado OR cuidado personal OR autoayuda | 124 |

LILACS PORTUGUESE

| Number | Search | Results |
| --- | --- | --- |
| #1 | Mal de Hansen OR hanseníase OR doença de Hansen | 2.590 |
| #2 | Auto cuidado OR cuidado pessoal OR auto ajuda | 403 |
| #4 | Mal de Hansen OR hanseníase OR doença de Hansen AND Auto cuidado OR cuidado pessoal OR auto ajuda | 4 |

SCIELO PORTUGUESE

| Number | Search | Results |
| --- | --- | --- |
| #1 | hanseníase OR doença de Hansen | 271 |
| #2 | Autocuidado OR cuidado pessoal OR auto ajuda | 1490 |
| #4 | hanseníase OR doença de Hansen AND Autocuidado OR cuidado pessoal OR autoajuda | 119 |

SCIELO SPANISH

| Number | Search | Results |
| --- | --- | --- |
| #1 | lepra OR enfermedad de Hansen OR hanseniasis | **5 208** |
| #2 | Autocuidado OR cuidado personal OR autoayuda, | **1 247 342** |
| #4 | Mal de Hansen OR lepra OR enfermedad de Hansen AND Autocuidado OR cuidado personal OR autoayuda | 12 |
