# Supplementary material for: Self-care interventions to reduce, prevent or monitor physical disability in those affected by leprosy: Protocol for a systematic review
Source: PLoS One. 2025 Oct 30;20(10):e0330477. doi: 10.1371/journal.pone.0330477 (PMC12574909; doi:10.1371/journal.pone.0330477)
Supplement: S3 File — (DOCX) [file pone.0330477.s003.docx]

| **Author and Title** | **Study Methodology/ Design** | **Country/ Region and setting** | **Population/ Sample** | **Self-care methods investigated** | **Reported outcomes** | **What did the researchers conclude** | **Other Relevant Data** |
| --- | --- | --- | --- | --- | --- | --- | --- |
|  |  |  |  |  |  |  |  |
